# Unraveling Pectin–Protein Interactions in Milk-Based Cocoa Beverages: Molecular Docking and Microstructure

**DOI:** 10.3390/foods15203697

**Published:** 2026-10-17

**Authors:** Saniye Akyıl Öztürk, Arezou Habibzadeh Khiabani, Deniz Damla Altan Kamer, Ali Can Karaca, Nevzat Konar, Senay Simsek, Omer Said Toker

**Affiliations:** 1 Chemical and Metallurgical Engineering Faculty, Food Engineering Department, Yildiz Technical University, Istanbul 34220, Türkiye; 2 Azelis Application & Training Center, Istanbul 34771, Türkiye; 3 Agriculture Faculty, Food Engineering Department, Tekirdag Namik Kemal University, Tekirdag 59030, Türkiye; 4 Electrical-Electronics Faculty, Computer Engineering Department, Yıldız Technical University, Istanbul 34220, Türkiye; 5 Agriculture Faculty, Dairy Technology Department, Ankara University, Ankara 06110, Türkiye; 6 Whistler Center for Carbohydrate Research, Department of Food Science, Purdue University, West Lafayette, IN 47907, USA

**Keywords:** pectin, beverage, cocoa, microstructure, milk, molecular docking

## Abstract

This study investigated the synergistic effects of pectin concentration (0.038–0.462%) and homogenization pressure (5.85–34.1 MPa) on the physical and microstructural stability of milk-based cocoa beverages. A Central Composite Rotatable Design (CCRD) was employed to optimize these parameters by evaluating critical quality attributes, including water-holding capacity (WHC), sedimentation, creaming index, apparent viscosity, and colloidal stability. To elucidate the underlying stabilization mechanisms, molecular docking simulations were integrated to assess the thermodynamic binding affinities between pectin (D-galacturonic acid) and major milk proteins. Numerical optimization identified 0.40% pectin and a homogenization pressure of 10 MPa as the optimal formulation and processing conditions. Under these conditions, the beverage achieved a maximum WHC of 86.04%, complete elimination of macroscopic phase separation (n.d. sedimentation and creaming), and an apparent viscosity of 67.39 mPa·s. Mechanistically, molecular docking revealed strong, multifaceted hydrogen-bonding and electrostatic interactions between pectin and milk proteins, with β-lactoglobulin exhibiting the strongest predicted thermodynamic binding affinity (−6.3 kcal/mol). This macromolecular complexation promoted pronounced steric and electrostatic stabilization, as supported by the highly negative zeta potential (−30.8 mV) and enhanced microstructural uniformity observed through scanning electron microscopy–energy-dispersive X-ray spectroscopy (SEM–EDS) analysis. A rigorous 9-month storage study further demonstrated prolonged kinetic stability. Structural integrity was largely maintained, and macroscopic phase separation was substantially delayed despite time-dependent rheological rearrangements during storage. Furthermore, sensory profiling demonstrated high consumer acceptability, particularly for smoothness and homogeneity, with the optimized formulation outperforming the evaluated commercial counterparts. Collectively, these findings demonstrate the effectiveness of pectin in such beverage system and provide practical formulation and processing strategies for improving the physicochemical stability and shelf life of complex dairy-based dispersions.

## 1. Introduction

Beverage stability refers to the ability of a beverage to resist phase separation and sedimentation over time. Flavored milk represents a promising approach for increasing milk consumption because of its nutritional value, sensory appeal, and desirable consistency. However, maintaining physical stability remains a major technological challenge for the dairy industry. Stability is particularly important in milk-based beverages, which are widely consumed but highly susceptible to physical destabilization [1,2]. These beverages are complex colloidal systems composed of milk proteins, fat globules, cocoa particles, and dissolved carbohydrates. Cocoa powder being rich in insoluble components leads to problems such as phase separation in water-based systems. In order to overcome the issue of cocoa solubility, the alkalization and agglomeration processes can be employed for increasing solubility of cocoa. The alkalization of cocoa modifies its color, flavor, aroma, and functional characteristics—especially solubility—based on factors like the kind of alkali used, its concentration, and processing conditions such as temperature and pressure [3]. Moreover, the inherent density differences between cocoa particles and the continuous milk phase can lead to sedimentation, creaming, and phase separation during processing and storage, thereby reducing product quality and consumer acceptance [2,4]. Sedimentation problems primarily arise from insufficient hydrocolloid usage, inappropriate hydrocolloid selection for the system, excessive cocoa powder particle size, reduced fat and protein content, and elevated filling temperatures.

Cocoa-based beverages are favored not only for their sensory appeal but also for their convenience in preparation and consumption [5]. Consumer preference for milk-based cocoa beverages is strongly associated with viscosity, which is influenced by protein and fat content, type and amount of hydrocolloids used in the formulation and the colloidal stability of the beverage [1]. Therefore, improving the stability of cocoa beverages is important for enhancing consumer acceptance, commercial value, and overall product quality. Improvements in physical stability may be particularly important for milk-based cocoa beverages because consumer perception is closely linked to their rheological and colloidal characteristics. The continued need to optimize both formulation and processing conditions has stimulated the development of new stabilization strategies.

To date, studies investigating stabilizer optimization in milk-based cocoa beverages have largely focused on hydrocolloids and other natural stabilizers already widely used in the food industry. Several stabilizer/emulsifier systems (lecithin, inulin, Carrageenan and k-carrageenan, gellan gum, carboxymethyl cellulose/carrageenan/gellan gum) are used to reduce sedimentation of cocoa in this type of beverages [6]. Among the hydrocolloids used in chocolate milk beverages, kappa-carrageenan is the most frequently used, because of the unique properties of carrageenan/milk mixtures due to the interaction of carrageenan molecules with casein micelles [7,8,9]. However, the potential application of other naturally derived stabilizers, such as citrus-derived pectin, warrants further investigation. Pectin is a structurally heterogeneous carbohydrate-based polysaccharide composed of repeating saccharide units. Its macromolecular structure primarily contains three major structural domains and is characterized by chains rich in methyl-esterified D-galacturonic acid residues. The molecular characteristics of pectin are strongly influenced by factors such as the plant source, extraction method and conditions, storage, and subsequent processing treatments [10,11].

Pectin is predominantly located in the primary cell wall and middle lamella of dicotyledonous plants and is one of the most widely utilized hydrocolloids in food systems. Owing to its distinctive structural and functional properties, pectin is used in a broad range of food applications, including jams, jellies, low-calorie products, and acidified milk beverages, as well as a thickening, stabilizing, and emulsifying agent [10]. In beverage manufacturing, pectin is generally incorporated at an appropriate stage of processing to promote effective hydration, dispersion, and stabilization. However, inappropriate timing or conditions of pectin incorporation may adversely affect its functionality and result in processing or stability problems.

Previous studies have demonstrated the effectiveness of hydrocolloids in improving the physical stability of milk-based beverages. Yuliarti et al. [12] reported that increasing the proportion of high-methoxyl pectin in combination with carboxymethyl cellulose significantly reduced serum separation and improved the physical stability of acidified milk drinks. Similarly, Akyil et al. (2025) [13] showed that the incorporation of citrus fiber improved the stability of cocoa-based dairy beverages by reducing sedimentation and increasing viscosity during storage. In addition, Akyıl Öztürk et al. [4] investigated the synergistic effects of carrageenan concentration and homogenization pressure on cocoa milk beverages. They demonstrated that increasing carrageenan concentration enhanced WHC, reduced sedimentation and creaming, and increased beverage viscosity.

In addition to stabilizer system in the formulation, production process is also important for obtaining the end product with desired quality. Ultra-high pressure homogenization (UHPH) has been introduced as a method to produce flavored milk with better hygienic preservation and colloidal stability compared to conventional heat treatments. During UHPH, the liquid is subjected to pressures between 100–400 MPa and forced at very high velocities through a narrow-gap valve using a high-pressure piston pump. This rapid flow generates physical phenomena such as cavitation, turbulence, impact, and shear forces, which act on suspended particles (like coffee or cocoa), cells, and macromolecules in the milk. These mechanical effects contribute both to the formation of fine, stable colloidal dispersions and to microbial inactivation [6,14]. UHPH enhances the solubility of functional ingredients and stabilizes emulsions without the need for high temperatures. It reduces droplet size and improves protein solubility in flavored milk [15]. High pressure homogenization also disrupts casein micelles and decreases polysaccharide molecular weight through pressure and shear forces, fostering hydrophobic interactions between proteins and polysaccharides that result in stable nano-scale aggregates in flavored milk [14]. Within the framework mentioned above, the stabilizer concentration and homogenization pressure used for producing cocoa-based beverages of the desired quality should be considered together. Taking this combination into account, optimization in terms of both formulation and process is highly important.

Although the effects of stabilizers on dairy beverage stability have been widely investigated, the combined influence of pectin concentration and homogenization pressure on the stability of milk-based cocoa beverages has not been systematically evaluated. In the present study, GENU^®^ YM-115-H is a citrus-derived, high-ester pectin grade intended for protein stabilisation in dairy systems. Therefore, this study addressed three main objectives: (1) to evaluate the effect of pectin concentration on the physical stability of milk-based cocoa beverages and provide further insight into its stabilizing mechanisms; (2) to investigate the influence of processing conditions, particularly homogenization pressure, on pectin functionality and its effectiveness as a stabilizer; and (3) to optimize formulation and processing conditions according to the desired physicochemical and quality characteristics of the final beverage.

## 2. Material and Methods

### 2.1. Materials

Pasteurized milk (3% fat, 3% protein) and granulated sugar were purchased from a local market. Pectin (Genu^®^ YM-115-H) extracted from citrus peel (high-ester pectin, about DE value of 72%, at least 65% galacturonic acid, non-amidated) was kindly supplied by CP Kelco (Lille Skensved, Denmark). Alkalized cocoa powder (10–12% fat, dark brown color, strongly alkalized; pH ≈ 8.80) was obtained from Altinmarka (İstanbul, Turkey). Dipotassium phosphate and potassium sorbate were supplied by Budenheim (Budenheim, Germany) and Nantong (China), respectively. All other chemicals used in this study were of analytical grade and purchased from Sigma-Aldrich Chemie (Steinheim, Germany).

### 2.2. Production of Milk-Based Cocoa Beverage and Experimental Design

A dry mixture consisting of sugar (6.00%), alkalized cocoa powder (1.00%), dipotassium phosphate (0.50%), potassium sorbate (0.05%), and pectin (concentration was determined using CCRD experimental design) was prepared. The pectin concentration range used in the study plan was determined by taking into account the recommendations of pectin- and cocoa-based beverage manufacturers. Normally, gellan gum and carrageenan are used in such beverages at concentrations of approximately 0.02–0.03%. Considering the viscosity provided by these thickeners to the product, the amount of pectin was determined accordingly. Based on preliminary trials and the suggestions of manufacturing companies, the range of pectin that could be used in the formulation was set to between 0.3–0.4%. However, in order to observe the effects of both lower and higher concentrations, the experimental design was prepared with a concentration range of 0.038% to 0.462%. The dry blend was added to the pasteurized milk, and the mixture was homogenized at 450 rpm using a Thermomix TM6 (Vorwerk, Wuppertal, Germany). The formulation was heated to 75 °C and immediately homogenized (Hommak C-HM1, İzmir, Turkey) at CCRD-specified pressures of 5.8–34.1 MPa (Table 1). After homogenization, the sample was held in a Thermomix TM6 at 95 °C for 4 min. The system was then rapidly cooled to 25 °C in a water bath (Nukleon NBS-30) and filled into glass vials sealed with plastic caps. Milk-based cocoa beverages were prepared under standardized processing conditions, consistent with industrial dairy production [16].

### 2.3. Water Holding Capacity (WHC)

WHC of the samples was assessed by centrifugation (Nuve, NF 400, Istanbul, Turkey) of a 50 mL sample at 4000 rpm for 10 min at room temperature. The volume of separated serum was measured in mL (Equation (1)) [17];

(1) $$\mathrm{WHC}\;(\%)=\left(1-\frac{\mathrm{Released}\;\mathrm{Serum}\;\mathrm{Volume}}{\mathrm{Original}\;\mathrm{Sample}\;\mathrm{Volume}}\right)\times 100$$

### 2.4. Sedimentation

Prepared beverages were stored at 4 ± 1 °C for 21 days. The sedimentation value (%) was calculated as the ratio of the sediment height at the bottom to the total height of the milk-based cocoa beverage.

(2) $$\mathrm{Sedimenation}\;\left(\%\right)=\frac{H_{S}}{H_{T}}*100$$

where *H_S_* is the height of the sediment and *H_T_* is the total height of the beverages

### 2.5. Creaming Index (%)

Prepared milk-based cocoa beverages were transferred to a glass bottle closed with a plastic cap and stored at 4 ± 1 °C for 21 days. The prepared beverages were filled into glass bottles and stored at 4 ± 1 °C for 21 days. Phase separation into two layers was observed during storage. The creaming index (CI) was determined using the following Equation (3);

(3) $$\mathrm{CI}\;\left(\%\right)=\frac{H_{C}}{H_{T}}*100$$

where *H_C_* is the height of the cream layer (top) and *H_T_* is the total height of the milk-based cocoa beverages.

### 2.6. pH

pH values of samples were determined at 25 °C, 24 h after production using pH-meter at 25 °C.

### 2.7. Viscosity (mPa·s)

Viscosity was measured using a viscometer (Lamy B-One Plus, Champagne au Mont d’Or, France) at 25 °C and 100 rpm. Measurements were recorded 2 min after the spindle was immersed (No. R-2) to ensure thermal equilibration of the sample. All measurements were performed in triplicate on the same sample.

### 2.8. Color

Color parameters were measured using a digital colorimeter (Konica Minolta Chroma Meter CR-410, Tokyo, Japan). According to the CIE L*a*b* color space, L* represents lightness, +a* and −a* indicate redness and greenness, and +b* and −b* indicate yellowness and blueness, respectively.

### 2.9. Zeta Potential Value and Particle Size

The optimum formulation was diluted 100-fold with 5 mM phosphate buffer. The ζ-potential of the diluted milk-based cocoa sample was measured at 25 °C using a Zetasizer Nano ZS90 (Malvern Instruments Ltd., Malvern, UK). Measurements were performed in triplicate. Droplet size distribution was determined using a Mastersizer 2000 (Malvern Instruments Ltd., UK). 

### 2.10. Microstructural Characterization

To evaluate the spatial organization and internal network architecture of the formulated milk-based cocoa beverages, comprehensive microstructural analyses were conducted. Initially, to preserve the delicate colloidal matrix without inducing structural collapse, the beverage samples were frozen at −20 °C and subsequently lyophilized for 48 h utilizing a vacuum freeze-dryer (Liyolife FD5CT, Manisa, Turkey). Following sample preparation, the microstructures of the beverages were extensively analyzed employing a JEOL JIB-4601F MultiBeam FIB-SEM system (Tokyo, Japan) to perform high-resolution microstructural imaging and elemental mapping using energy-dispersive X-ray spectroscopy (EDS). Furthermore, supplementary electron micrographs were acquired using a Zeiss EVO^®^ LS 10 scanning electron microscope (Jena, Germany) at specific magnifications of 10× and 40× to capture broader structural perspectives.

Beyond qualitative observations, a rigorous quantitative evaluation of the image features was executed utilizing a gray-level co-occurrence matrix (GLCM) coupled with a local window analysis procedure meticulously implemented in MATLAB 2023b (USA). To systematically describe the microstructure characteristics, non-overlapping W × W spatial patches were extracted from each acquired image, where ‘W’ denotes the designated local window size. From each isolated patch, specific statistical and quality features were calculated. This systematic variation in the W value was crucial to evaluate its influence on the extracted features, thereby enabling highly robust and objective comparisons among the different formulations. Evaluation metrics extracted from these non-overlapping patches on the SEM images were thoroughly assessed to quantify quality shifts.

The primary complementary descriptors extracted from the GLCM analysis encompassed entropy, variance, and homogeneity. In this analytical context, entropy served to quantify the degree of randomness within each defined patch, thereby accurately reflecting the overall quality complexity and structural disorder of the matrix. Variance was computed to describe the specific dispersion of pixel intensities localized around the mean, which functioned as an indicator of local structural contrast and particle clustering. Concurrently, homogeneity evaluated the uniformity of the spatial gray-level distribution, effectively reflecting the consistency and structural cohesion of the samples. By leveraging the advanced computational capabilities of MATLAB to analyze multiple varying patch sizes and compute these sophisticated quality descriptors, this methodology provided an exceptionally comprehensive characterization of the microstructural and quality attributes of the colloidal beverage systems.

### 2.11. In Silico Molecular Docking Simulations

To elucidate the specific binding mechanisms and thermodynamic affinities between pectin and primary milk proteins at the molecular level, in silico molecular docking simulations were executed. The three-dimensional (3D) crystallographic structures of the whey proteins, specifically α-lactalbumin (α-LA, PDB ID: 6IP9) and β-lactoglobulin (β-LG, PDB ID: 3NPO), were retrieved from the RCSB Protein Data Bank. Prior to docking, the macromolecular targets were computationally purified by stripping water molecules and non-essential ligands using UCSF Chimera to isolate the active binding sites. Due to the lack of complete crystallographic data for the casein fractions, the 3D structures of αs1-casein (αs1-CN) and β-casein (β-CN) were generated via homology modeling utilizing the I-TASSER server. The dominant structural monomer of pectin, D-galacturonic acid (D-GalA), was selected as the ligand; its structure was retrieved from the PubChem database and geometrically optimized using ChemDraw 26.0. The molecular docking algorithms were executed utilizing AutoDock Vina v1.2.7 (PyRx platform) to conduct virtual screening and calculate the binding energies. The resulting protein–ligand conformations were systematically visualized, and specific non-covalent interactions were mapped in 2D and 3D spaces using BIOVIA Discovery Studio 2022, ProteinsPlus (DoGSiteScorer, Hamburg, Germany), and the Protein & Ligand Interaction Profiler (PLIP). The simulations were conducted under the boundary assumption that interactions were exclusively binary (protein & pectin), thereby isolating these specific binding events from potential competitive interference by other matrix constituents.

### 2.12. Storage Stability

Samples produced under optimal conditions were stored for 9 months at 4.00 ± 1.00 °C. During storage, WHC, sedimentation, creaming index (CI), pH, and viscosity were measured at 30 days intervals. All analyses were performed at defined time points, and the data were statistically analyzed.

### 2.13. Sensory Analysis

Sensory evaluation was conducted by a panel of 10 trained chocolate and beverage technology experts (4 males, 6 females; 20–32 years). A 5-point hedonic scale was used for the sensory analysis, which included smoothness, mouthfeel (texture and creaminess), homogeneity, flavor, and overall acceptability.

### 2.14. Statistical Analysis

Results are presented as mean ± standard deviation. The effects of independent factors on response variables were evaluated by analysis of variance (ANOVA), followed by Tukey’s post hoc test for pairwise comparisons among samples. All statistical analyses were performed using SPSS software (IBM SPSS Statistics 22, USA) at a significance level of *p* < 0.05. The response surface methodology and optimization was carried out by Design Expert (Design-Expert 13, USA).

## 3. Results and Discussion

### 3.1. Physico-Chemical Properties of Milk-Based Cocoa Beverages

**Figure 1:**
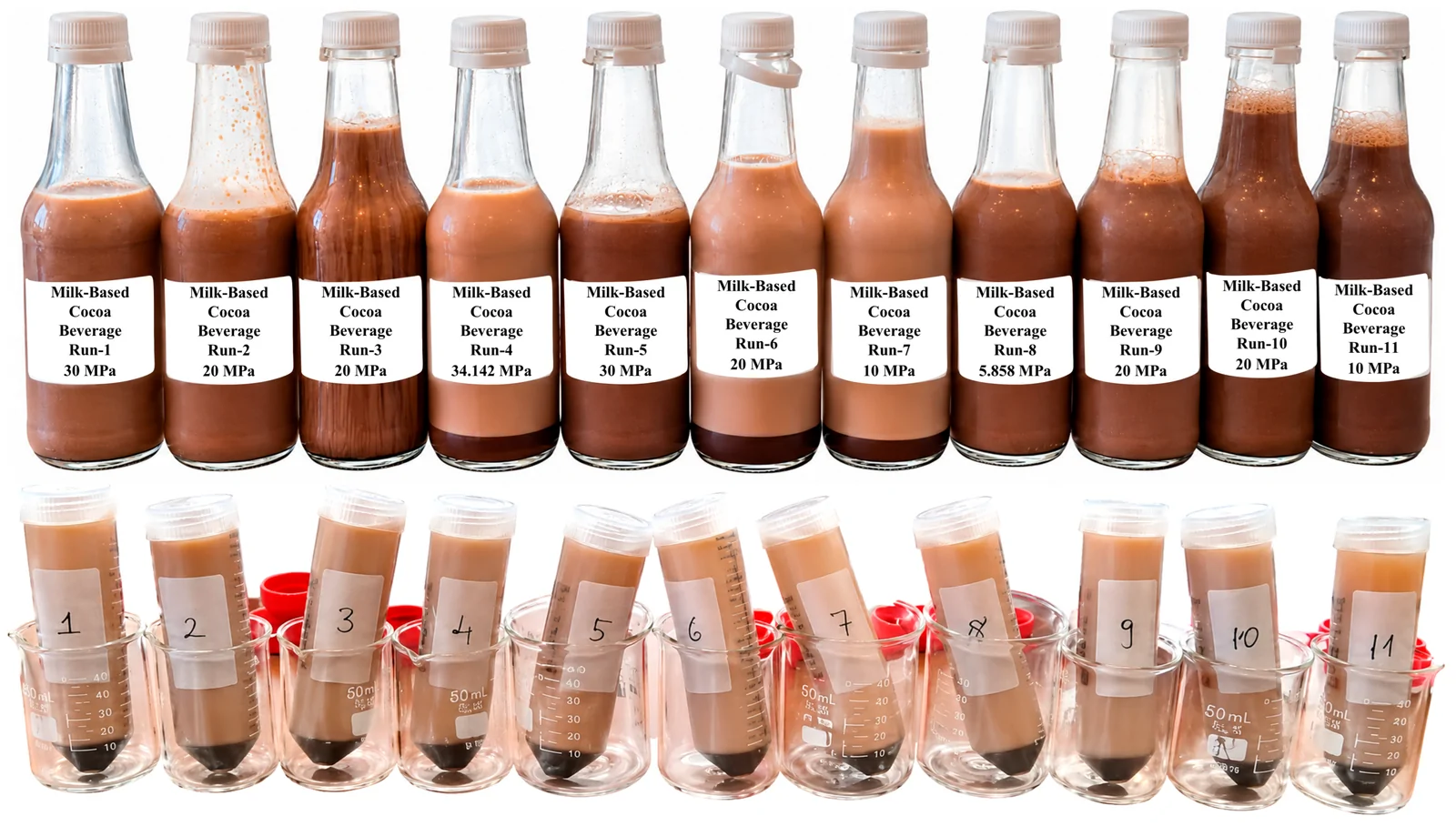
Images of milk-based cocoa beverages produced according to the CCRD experimental design. From left to right: Run-1 to Run-11. Table 1 provides detailed information on the production process.

The physicochemical attributes of the milk-based cocoa beverages, formulated under varying processing conditions guided by the CCRD, are summarized in Table 1. Furthermore, their macroscopic phase separation behaviors following storage are visually depicted in Figure 1.

**Figure 2:**
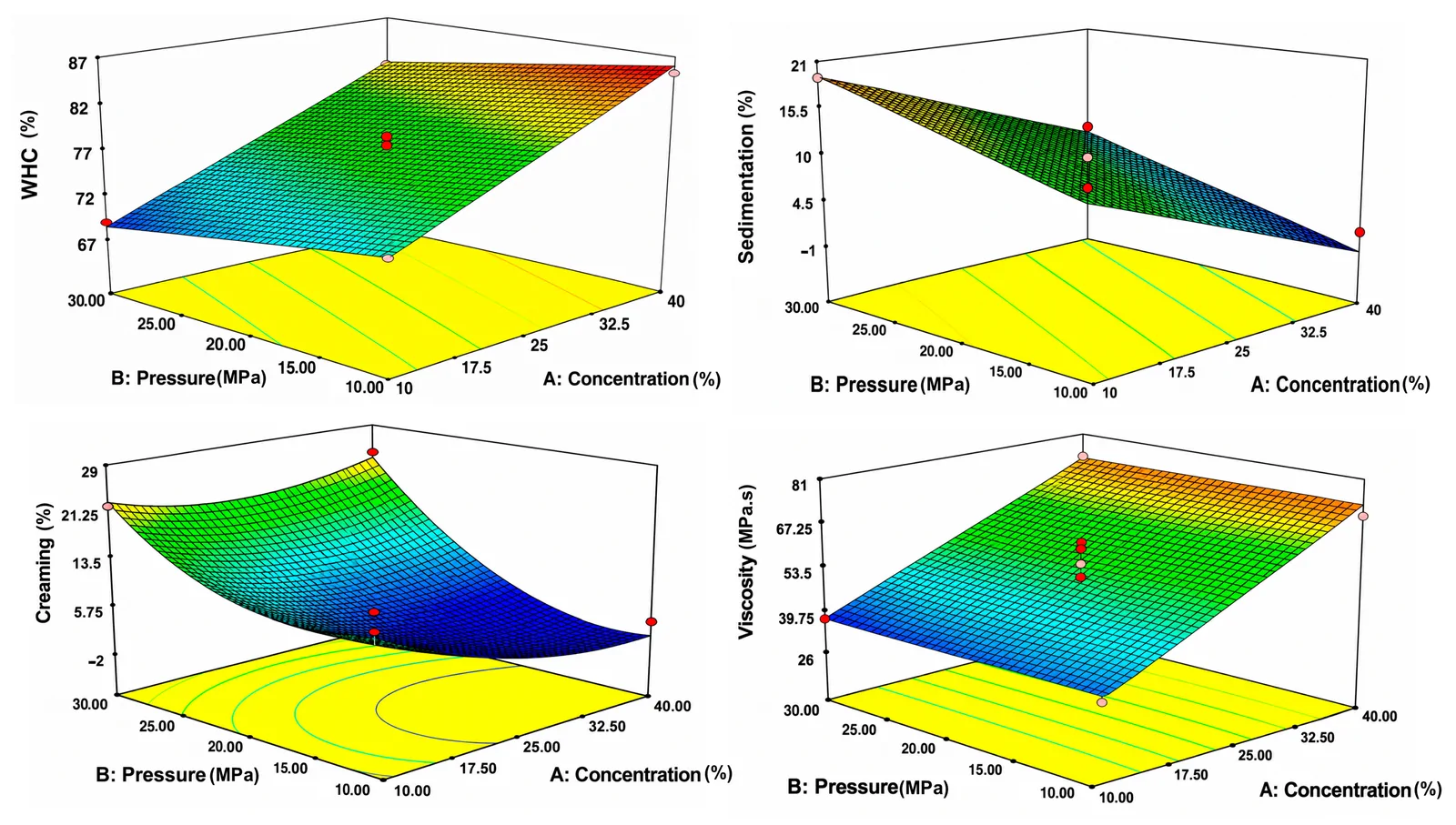
Effects of pectin concentration and homogenization pressure parameters on dependent parameters of the sample.

The WHC, defined as the amount of water retained relative to the incorporated pectin, varied substantially among the 11 experimental runs, ranging from 67.00% to 86.50%. The highest WHC (86.50 ± 0.71%) was observed in Run 2, which contained a high pectin concentration (0.462%) and was homogenized at 20 MPa. Results highlighted that WHC increased with homogenization pressure until some level. The increase in WHC following high-pressure homogenization may be attributed to the breakdown of pectin aggregates and the resulting increase in surface area, which promotes greater water retention within the pectin network [18]. Excessively high homogenization pressure may disrupt the pectin structure, thereby reducing its WHC. In contrast, the lowest WHC (67.00 ± 0.99%) was recorded in Run 6, which contained 0.038% pectin at the same homogenization pressure. As illustrated by the three-dimensional response surface plots (Figure 2), increasing pectin concentration had a pronounced positive effect on WHC, whereas increasing homogenization pressure tended to reduce this parameter. Although the regression model developed for WHC was not statistically significant (*p* > 0.05), the high coefficient of determination (*R*^2^ = 0.9871) indicated close agreement between the experimental and model-predicted values. Nevertheless, because the overall model was not statistically significant, the response surface trends should be interpreted with appropriate caution. These findings are consistent with those of Akyil et al. [13], who demonstrated that hydrocolloid supplementation, including citrus fiber, can enhance water retention and structural stability in cocoa-based dairy beverages. Mechanistically, pectin can promote water immobilization within dairy matrices because of its high water-binding capacity and ability to form hydrated polymeric networks that restrict water mobility and enhance the structural integrity of the system [19]. The free carboxyl groups present in the pectin structure can form hydrogen bonds with water molecules, thereby retaining water within the structure and resulting in an increase in WHC [20].

The creaming index, another important indicator of phase separation in dairy-based dispersions, ranged from 1.43% to 28.27% across the experimental runs. Analysis of variance (ANOVA) indicated that the response could be described by a quadratic model with a high coefficient of determination (*R*^2^ = 0.9747), demonstrating a strong relationship between the experimental values and model predictions. The highest creaming index (28.27 ± 0.82%) was observed in Run 4, corresponding to a pectin concentration of 0.25% and the highest homogenization pressure of 34.1 MPa. Conversely, the lowest creaming index (1.43 ± 0.06%) was recorded in Run 8, which contained 0.25% pectin and was homogenized at the lowest pressure of 5.85 MPa. Homogenization results in smaller fat globules, which contribute to improved emulsion stability and reduced creaming tendency [21]. Fat globules of greater size rise more rapidly, causing higher creaming levels. This dimension is affected by milking conditions, with suboptimal procedures or machine adjustments leading to enlarged globules and more evident creaming [21,22]. More oil droplets on the beverage surface due to higher homogenization pressure levels result in higher viscosity and a creamier texture [23]. Higher viscosity within the dairy medium restricts fat globule motion and consequently reduces creaming [21]. The experimental results suggest that excessive homogenization pressure, particularly when stabilizer concentration is insufficient relative to the newly generated interfacial area, may promote creaming rather than improve physical stability. One possible explanation is an over-homogenization effect, in which intense mechanical shear produces a large population of smaller droplets and substantially increases the total interfacial area. If sufficient pectin or other surface-active components are not available to stabilize these newly formed interfaces, incomplete surface coverage may promote droplet–droplet interactions, flocculation, and the formation of larger buoyant aggregates, thereby accelerating creaming. Moreover, thermal processing and additional treatments such as mechanical forces applied during homogenization may modify the structures of fats and proteins, thereby affecting the creaming characteristics [24]. For this reason, the different pressure levels applied during homogenization have led to variations in the creaming index value of the beverages.

Evaluation of pH demonstrated that variations in pectin concentration and homogenization pressure had no statistically significant effect on this parameter (*p* > 0.05). The pH remained within a narrow range of 6.83–6.89 across the experimental treatments. These results indicate that neither pectin incorporation nor the applied homogenization conditions substantially altered the acid–base characteristics of the milk-based cocoa beverage. The limited variation in pH can largely be attributed to the intrinsic buffering capacity of milk, particularly that is provided by milk proteins, phosphate salts, citrate, and other ionic components. Consequently, the formulation and processing conditions evaluated in this study had minimal influence on the overall pH of the beverage.

The viscosity value of cocoa-based milk beverages plays a critical role in determining not only the sensory characteristics of products but also essential quality parameters such as creaming and sedimentation. It is simultaneously affected by a wide range of process- and formulation-based factors. Rheological analyses demonstrated that pectin concentration and homogenization pressure significantly influenced the viscosity value of the colloidal system (*p* < 0.05). Apparent viscosity across the experimental treatments ranged from 26.53 ± 0.50 to 80.68 ± 1.11 mPa·s. As indicated by the linear model (R^2^ = 0.9745) and the response surface presented in Figure 2, apparent viscosity increased significantly with increasing pectin concentration. Pectin, a heteropolysaccharide, contains many hydrophilic sites—primarily galacturonic acid residues—that capture and retain water through hydrogen bonds and electrostatic forces [25,26]. Through cross-linking of pectin molecules, calcium ions promote greater water binding, resulting in a firmer and more expansive gel structure [27]. In dairy-based systems, pectin, particularly at higher concentrations, can interact with casein micelles through adsorption and association at the micellar surface, contributing to the formation of a steric layer around the dispersed particles [28,29]. These interactions increase the effective hydrodynamic volume of the dispersed phase and restrict particle mobility, thereby increasing resistance to flow and, consequently, the apparent viscosity of the beverage. pH is the critical parameter for this interaction. At acidic pH, positively charged milk proteins and negatively charged pectin interact via electrostatic attraction. This mechanism can generate soluble or insoluble complexes, influenced by conditions such as ionic strength, pH, and the extent of pectin esterification [30,31]. Additionally, HPH treatment caused variation in the viscosity of the samples, which may be attributed to the reduction in particle size and the subsequent formation of a more homogeneous and stable suspension of cocoa and fat as well as structural modification of pectin and the other macromolecules such as protein and fat as a result of the HPH process [32].

Torque, which provides an additional measure of rheological behavior and resistance of the structural network to deformation, ranged from 0.119 ± 0.011 to 0.362 ± 0.005 mN·m. Torque followed a trend similar to that observed for apparent viscosity, increasing significantly (*p* < 0.05) with increasing pectin concentration. Increased homogenization pressure also positively influenced the torque response of the system, suggesting that processing intensity contributed to changes in the internal organization and resistance of the colloidal structure. These findings are consistent with previous studies demonstrating that increasing hydrocolloid concentration increases apparent viscosity by enhancing resistance to flow and promoting the development of more structured macromolecular networks [33].

### 3.2. Optimization

To maximize the physical stability of the milk-based cocoa beverages, a multiple-response optimization was performed utilizing the empirical data derived from the CCRD. The primary objective function of this optimization was to maximize the WHC while simultaneously minimizing the sedimentation and creaming indices. Concurrently, the apparent viscosity (70–90 mPa·s), torque (0.30–0.45 mN·m), and pH were constrained within acceptable industrial ranges to ensure optimal rheological and sensory attributes.

Employing the desirability function approach, the numerical optimization yielded a remarkably high overall desirability score of 0.994, indicating that the predefined optimization constraints were simultaneously achieved. Based on this analysis, the optimal processing parameters were established at a pectin concentration of 0.40% and a homogenization pressure of 10 MPa. Under these optimal conditions, the predictive model projected a WHC of 86.17%, sedimentation and creaming indices of 0.00%, an apparent viscosity of 69 mPa·s, and a torque value of 0.294 mN·m.

To rigorously validate the predictive capability and reliability of the formulated optimization model, at least three independent validation trials (*n* ≥ 3) were conducted under the determined optimal parameters. The experimental validation results, detailed in Table 2, exhibited a robust statistical concordance with the theoretically predicted values, with 86.04 ± 0.51% WHC, 67.39 ± 0.55 mPa·s viscosity, and 0.293 ± 0.014 mN·m torque value. This strong agreement definitively confirmed the reliability and accuracy of the CCRD optimization model. Consequently, this validated set of optimized processing conditions was selected as the reference formulation for subsequent advanced microstructural characterization, molecular docking analyses, and long-term storage stability evaluations.

### 3.3. Molecular Binding Mechanisms and Docking Simulations

**Figure 3:**
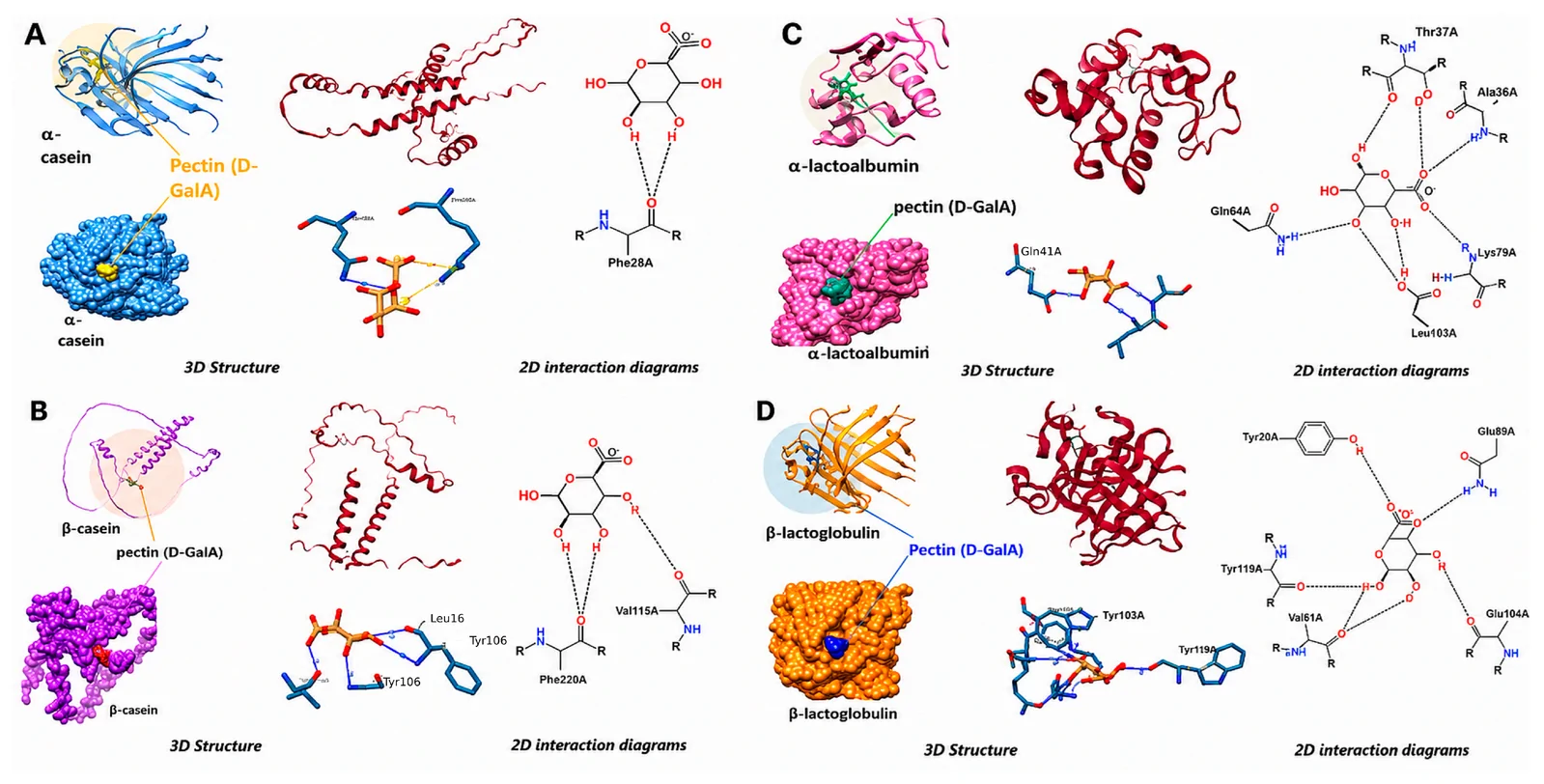
Molecular docking surface 3D structures, and 2-D interaction diagrams of α-casein (**A**), β-casein (**B**), α-lactoalbumin (**C**) and β-lactoglobulin (**D**), respectively.

Molecular docking simulations were performed to elucidate the potential binding mechanisms and relative affinities between D-galacturonic acid (D-galA), the predominant structural monomer of pectin, and the major milk proteins α-casein, β-casein, α-lactalbumin, and β-lactoglobulin (Figure 3 and Table 3). The docking analysis indicated that the interaction between α-casein and D-galA was mediated by a combination of hydrogen bonding and electrostatic interactions. One hydrogen bond was formed with Gln170 at an intermolecular distance of 2.76 Å. In addition, two salt bridges were identified between the positively charged Arg166 residue and the negatively charged carboxylate group of D-galA, with interaction distances of 4.13 and 4.49 Å, respectively. These electrostatic interactions may contribute to stabilization of the α-casein–D-galA complex. The predicted binding energy was −4.2 kcal/mol, suggesting a moderate affinity between D-galA and the identified binding region of α-casein.

In comparison, the interaction between β-casein and D-galA was characterized by a network of four hydrogen bonds. These interactions involved Val216 (2.00 Å), Gly218 (2.63 Å), and Phe220, which formed two hydrogen bonds at distances of 2.24 and 3.05 Å. The predicted binding energy of the β-casein–D-galA complex was −3.7 kcal/mol, representing the least favorable docking score among the proteins investigated. Nevertheless, the presence of multiple hydrogen bonds indicates the potential for localized interactions between D-galA and β-casein that could contribute to protein–pectin association within the beverage matrix.

For the whey protein fractions, α-lactalbumin exhibited three hydrogen-bonding interactions with D-galA involving Glu49 (2.16 Å), Leu105 (2.22 Å), and Ala106 (2.25 Å). These relatively short intermolecular distances indicate close association between polar functional groups of D-galA and accessible regions of the protein surface. The predicted binding energy was −5.3 kcal/mol, indicating a more favorable interaction than those observed for α- and β-casein. In particular, the short hydrogen-bonding distance associated with Glu49 suggests that this residue may play an important role in stabilizing the docked α-lactalbumin–D-galA complex.

Among the milk proteins evaluated, β-lactoglobulin exhibited the most favorable predicted binding affinity toward D-galA, with a docking energy of −6.3 kcal/mol. The β-lactoglobulin–D-galA complex was stabilized by an extensive network of seven predicted hydrogen bonds involving Trp19 (2.28 Å), Tyr42 (2.76 Å), Gln59 (2.20 and 3.48 Å), Cys160 (3.23 Å), and His161 (3.17 and 2.71 Å). The greater number of hydrogen-bonding interactions, together with the more favorable docking score, suggests stronger molecular association of D-galA with β-lactoglobulin relative to the other milk proteins examined. Collectively, the docking results demonstrate that D-galA is capable of interacting with both casein and whey proteins through hydrogen bonding and, in the case of α-casein, salt-bridge formation. Such molecular interactions may contribute to the association between pectin and milk proteins and thereby help explain the enhanced physical stability observed in the optimized milk-based cocoa beverage system.

Pectin–milk protein interactions are primarily driven by pH-dependent electrostatic attraction or repulsion, and the nature of the proteins—casein micelles versus whey proteins—determines the result. Depending on system parameters, pectin can act as a stabilizer by adsorbing onto protein surfaces or trigger flocculation and phase separation when polymer bridging or exclusion occurs [34,35,36]. When considering pH value of milk-based cocoa beverages, around neutral pH, pectin primarily interacts with casein micelles in milk. At pH 6.7, the main mechanism is depletion flocculation, caused by the exclusion of pectin between micelles, which indirectly enhances their attraction. At more acidic conditions (≤5.3), pectin attaches to micelle surfaces, with low levels leading to bridging flocculation and higher levels producing full coverage that diminishes attraction [34]. As a summary, the effect of pectin on milk proteins varies with system parameters like pH, ionic strength, and esterification level. For casein micelles, pectin usually stabilizes acidic milk by adsorbing onto their surfaces, creating coatings or weak networks, while close-to-neutral pH can lead to depletion flocculation and phase separation. For whey proteins, pectin often engages in electrostatic complexation, which can enhance stability, gelation, or emulsifying properties when conditions are suitable [34,35,36]. These interactions are highly important for the viscosity, sedimentation, creaming, and shelf-life stability of the beverage.

### 3.4. Zeta Potential and Colloidal Stability of Beverages

The zeta potential of colloidal dispersions is a key indicator of their electrostatic stability and tendency to aggregate [4]. The zeta potential of the beverage averaged −30.80 mV (Table 4), indicating a stable colloidal system. In general, increasing the concentration of certain stabilizers, such as modified high-methoxyl pectin, in fermented milk beverages can increase the zeta potential. However, reducing the pH tends to decrease it [28]. Pectin can enhance zeta potential due to its negative charge and its ability to interact with positively charged components, such as proteins (e.g., lactoferrin or casein), primarily through electrostatic interactions and hydrogen bonds. Pectin enhances colloidal stability by increasing the negative surface charge and electrostatic repulsion between particles, thereby preventing aggregation. For instance, an optimized cocoa-based dairy beverage formulation utilizing citrus fiber as a stabilizer achieved a zeta potential of −27.90 mV. This value indicated improved electrostatic stability, with negatively charged polysaccharide structures in citrus fiber and increased surface charge, leading to reduced particle aggregation [13].

### 3.5. Microstructure

**Figure 4:**
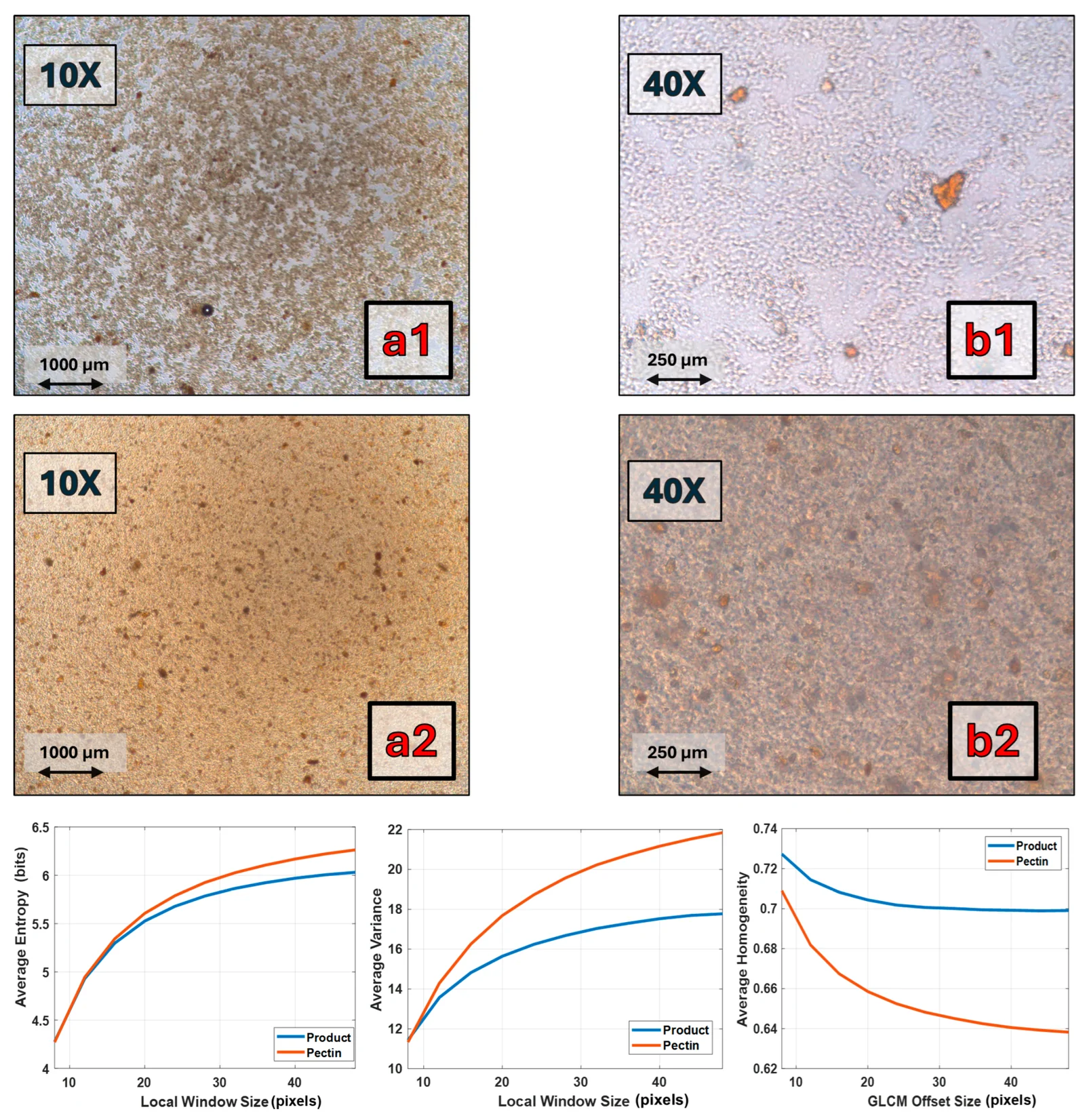
Microstructure of the optimum milk-based cocoa formulation (**a1**,**b1**) and a local market product (**a2**,**b2**) at 10× and 40× magnification. Scale bars represent 1000 µm and 250 µm, respectively. The bottom plots show texture metrics extracted from non-overlapping patches of the micrographs: average entropy in bits (**left**) and average variance (**middle**) as functions of local window size, and average gray-level co-occurrence matrix (GLCM) homogeneity (**right**) as a function of GLCM offset. Local window size and GLCM offset are expressed in pixels.

The microstructural characteristics of the milk-based cocoa beverages, evaluated through optical microscopy and subsequent quantitative image analysis, are illustrated in Figure 4. By employing a gray-level co-occurrence matrix (GLCM) and local window analysis, it was determined that the formulation at the optimum point exhibited the lowest entropy and variance across various window sizes. Because entropy serves as a quantitative metric for structural disorder, these diminished values substantiate that the optimized sample possessed a highly ordered, finely dispersed, and compact continuous network. The conspicuous reduction in particle aggregates implies that the optimized pectin concentration facilitated robust intermolecular interactions between the dispersed cocoa particulates and the continuous aqueous phase, thereby effectively mitigating flocculation and macroscopic phase separation. Conversely, the commercial local market product displayed the highest entropy and variance, revealing a markedly irregular structure characterized by larger, randomly distributed aggregates. This microstructural heterogeneity suggests comparatively weaker stabilizer & protein interactions and diminished colloidal stability within the commercial formulation. These findings unequivocally demonstrate that the synergistic optimization of hydrocolloid concentration and homogenization pressure fundamentally enhances microstructural uniformity, which translates to superior physical stability. This microstructural paradigm is consistent with the observations of [4,13], who reported that properly stabilized milk-based cocoa beverages exhibit significantly lower entropy and variance. These attributes reflect a homogeneous distribution of cocoa solids achieved via steric hindrance, which actively binds water and prevents particle sedimentation. In stark contrast, unoptimized or generic commercial formulations fail to provide such targeted microstructural support, resulting in pronounced structural disorder.

#### 3.5.1. Scanning Electron Microscopy (SEM)

**Figure 5:**
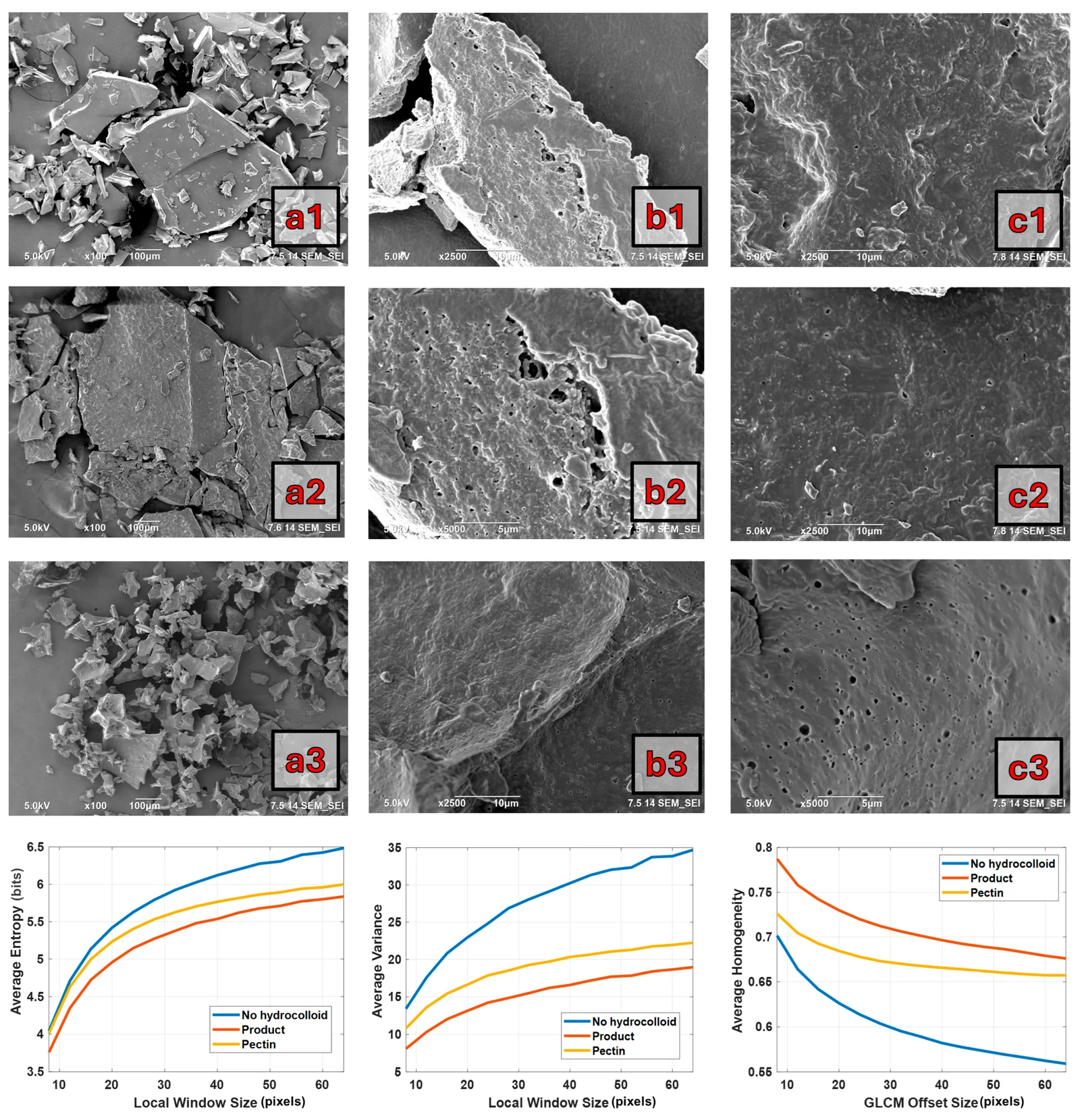
SEM images of milk-based cocoa beverages without hydrocolloid (**a1**–**c1**), at the optimum formulation (**a2**–**c2**), and from a local market product (**a3**–**c3**). Magnifications are indicated in each micrograph, and scale bars show the corresponding physical dimensions. The bottom plots present texture metrics extracted from non-overlapping patches of the SEM images: average entropy in bits (**left**) and average variance (**middle**) as functions of local window size, and average gray-level co-occurrence matrix (GLCM) homogeneity (**right**) as a function of GLCM offset. Local window size and GLCM offset are expressed in pixels.

To gain deeper insights into the surface topography and internal network structure of the samples, SEM was performed, with the resulting micrographs presented in Figure 5. The control sample, formulated without the addition of any hydrocolloid, exhibited a profoundly heterogeneous and disrupted structure across all evaluated magnifications (100×, 2500×, and 5000×). The prevalence of large, irregular voids and uneven topographical surfaces within this sample serves as a clear indicator of weak structural integrity and inherent colloidal instability. In striking contrast, the beverage formulated under the optimized conditions displayed a remarkably compact and homogeneous microstructural network across all magnifications. The substantial reduction in apparent pore size and the uniform spatial distribution of the structural components strongly suggest the formation of a well-organized, cohesive matrix. This enhanced structural organization is primarily attributed to the efficient adsorption of pectin onto the surfaces of milk proteins, coupled with the promotion of enhanced protein–polysaccharide interactions facilitated by the optimized homogenization pressure. As highly anionic polysaccharides, pectins function as potent stabilizing agents; their constituent molecules bind to the surface of milk proteins, thereby establishing a protective layer that inhibits aggregation. This mechanism, known as “steric stabilization,” effectively envelops the protein particles, creating a physical barrier that prevents close spatial contact and subsequently inhibits the spontaneous formation of larger aggregates [37]. Furthermore, the application of an optimized homogenization pressure plays a critical synergistic role by drastically refining the initial particle size and promoting uniform dispersion, thereby reinforcing the overall cohesion of the polymeric network. These microstructural enhancements are in excellent agreement with the macroscopic observations of improved WHC, elevated apparent viscosity, and the highly negative zeta potential recorded for the optimized sample. While the commercial product exhibited a relatively denser structural matrix compared to the hydrocolloid-free control, significant heterogeneity and localized aggregation were still visually evident. This indicates that although standard industrial formulations impart a baseline level of stabilization, the targeted optimization of pectin concentration and homogenization parameters achieved in this study yields vastly superior microstructural uniformity and stability. These empirical findings parallel those reported by [4,13], who noted that the strategic incorporation of carrageenan under optimized homogenization conditions drastically improved the microstructural uniformity of cocoa milk beverages, whereas conventional market products persistently displayed disordered and heterogeneous internal structures.

#### 3.5.2. Elemental Composition by Energy-Dispersive X-Ray Spectroscopy (EDS)

**Figure 6:**
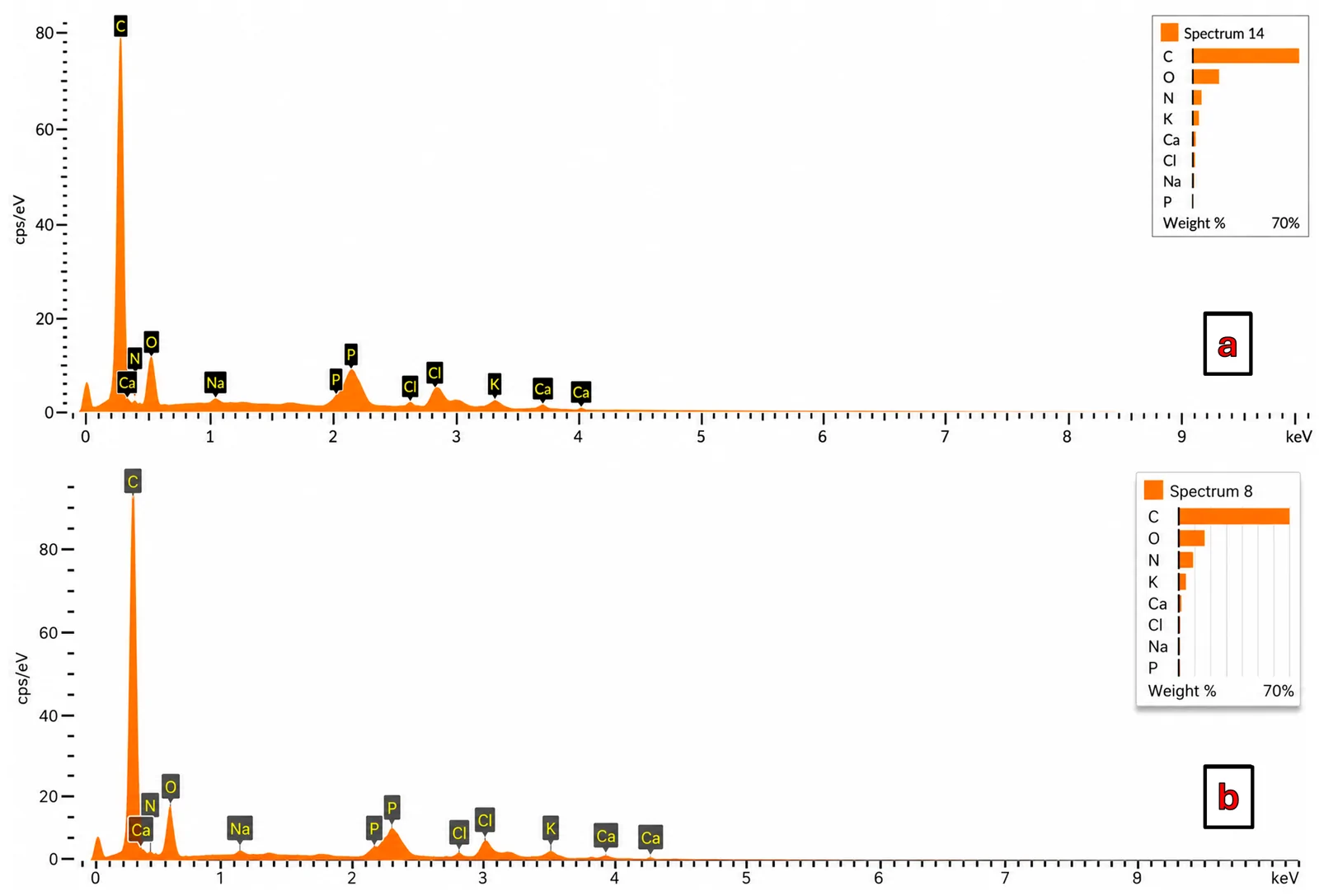
Energy-dispersive X-ray spectroscopy (EDS) spectra of milk-based cocoa beverages without hydrocolloid (**a**) and at the optimum formulation (**b**). The detected elemental peaks correspond to C, Ca, N, O, Na, P, Cl, and K.

Figure 6 and Table 5 illustrate the elemental composition of the samples, demonstrating the effect of pectin incorporation at the optimum point compared with the control formulation without a hydrocolloid. In both samples, carbon (C), oxygen (O), and nitrogen (N) were the dominant elements, reflecting the organic nature of the milk–cocoa matrix, which is composed of proteins, carbohydrates, and lipids. Minor peaks corresponding to Na, Cl, K, and Ca were also detected, which are associated with naturally occurring milk minerals and cocoa constituents. Results (Table 4) showed no significant differences (*p* > 0.05) in carbon, potassium, and nitrogen contents between the samples, indicating that the overall organic composition remained unchanged after formulation optimization. However, the optimum sample exhibited significantly higher phosphorus and oxygen content than the formulation without hydrocolloid (*p* < 0.05), which may be attributed to improved mineral distribution and enhanced functional interactions within the pectin–milk matrix. In addition, an increase in phosphorus content in the pectin-containing sample is attributed to microstructural reorganization of the milk matrix. In milk systems, phosphorus is primarily associated with colloidal calcium phosphate and phosphoserine residues within casein micelles. As an anionic polysaccharide, pectin can interact electrostatically with casein micelles, promoting micellar rearrangement and improving the retention and spatial distribution of phosphate-rich domains, thereby increasing the detectability of phosphorus in EDS analysis. Yuliarti et al. [12] investigated pectin adsorption onto casein micelles in acidified milk systems, showing the electrostatic stabilization mechanism of pectin at low pH. In contrast, sodium, chlorine, and calcium contents were significantly lower in the optimum sample (*p* < 0.05), suggesting a more homogeneous microstructure with reduced local mineral accumulation. Overall, the EDS results confirm that the optimization process did not alter the fundamental elemental composition but contributed to a more uniform distribution of inorganic components within the cocoa beverage system. Wilbanks [38] showed that pectin–casein interactions significantly alter the microstructure and stability of milk beverages. In the absence of pectin, casein micelles aggregate via protein–protein interactions, resulting in microstructural heterogeneity and clustering. In contrast, pectin interacts electrostatically with casein micelles via its anionic carboxyl groups, forming surface complexes that provide steric and electrostatic stabilization. This interaction hinders micellar aggregation, leading to a more homogeneous and stabilized microstructure.

### 3.6. Sensory Evaluation

**Figure 7:**
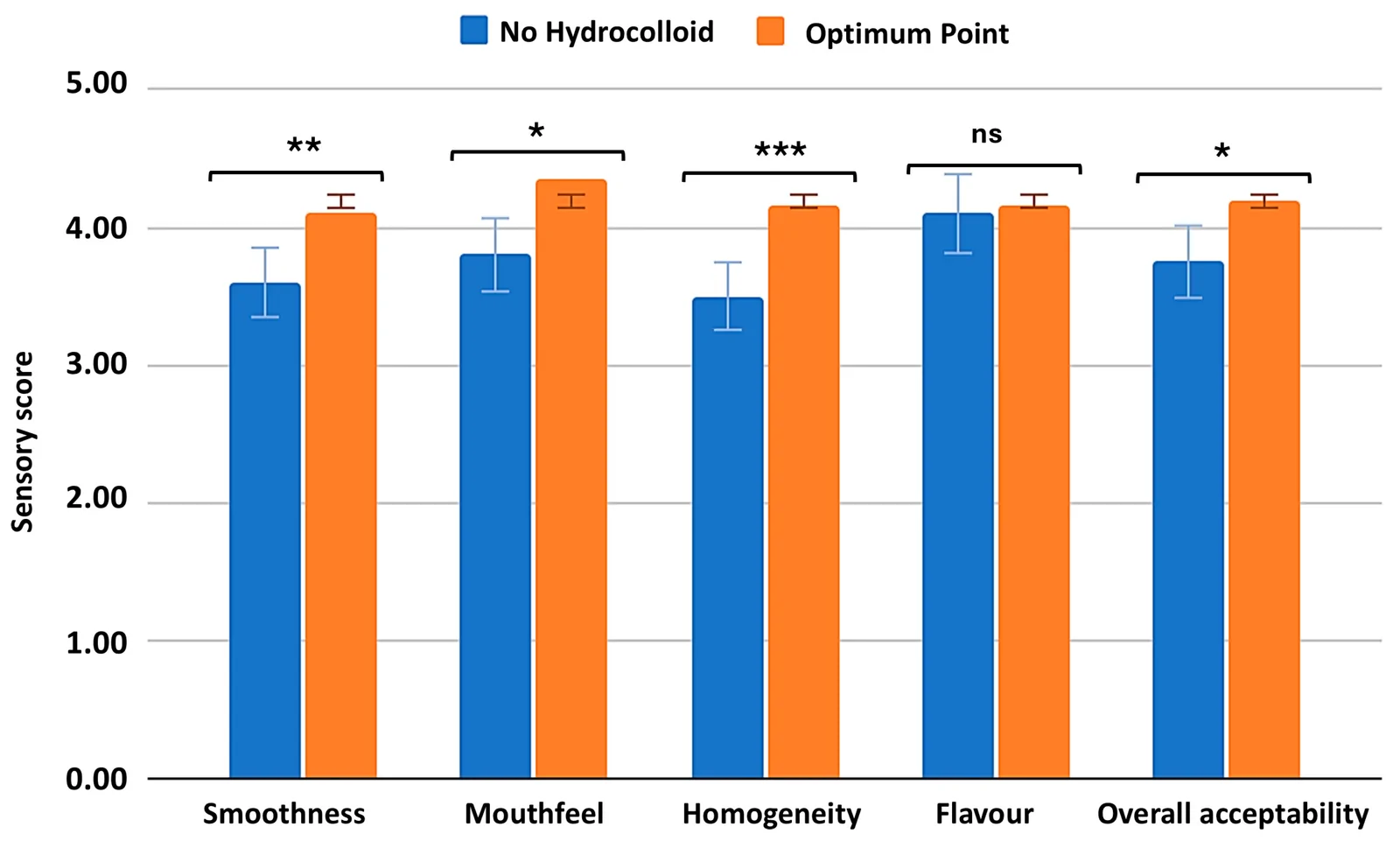
The sensory evaluation scores for without hydrocolloid and optimum point milk-based cocoa samples. (* *p* < 0.05, ** *p* < 0.01, *** *p* < 0.001, ns: non-significant).

The sensory evaluation of milk-based cocoa beverage samples is presented in Figure 7. As shown in Figure 7, the optimum formulation exhibited significantly higher sensory scores for smoothness, mouthfeel, homogeneity, and overall acceptability compared with the sample without hydrocolloid, indicating a more uniform and pleasant texture. No significant differences in flavor were detected in samples, suggesting that the hydrocolloid primarily affects physical attributes rather than altering the intrinsic taste profile of the cocoa beverage. The enhanced sensory performance of the optimum sample can be attributed to the hydrocolloid’s stabilizing effect on the beverage microstructure. Improved homogeneity and reduced particle aggregation contribute to a smoother texture and better mouthfeel, which, in turn, positively influence overall acceptability. This is in line with the findings of Costell et al. [39], who reported that reduced particle sedimentation contributes to a smoother texture and a more homogeneous structure, which is perceived as improved mouthfeel in cocoa beverages. The incorporation of citrus fiber likely enhanced mouthfeel and creaminess due to its high WHC and its capacity to form a gel-like network, which creates a lubricating effect during consumption [4]. In particular, EDS analysis showed a more uniform distribution of elemental components, suggesting improved compositional homogeneity, which is commonly associated with smoother texture and enhanced mouthfeel in dispersed cocoa beverage. However, hydrocolloid-free samples exhibited a rougher mouthfeel, likely due to sedimentation or inadequate particle suspension, a common limitation in unstabilized cocoa milk formulations. The sensory perception of increased smoothness and homogeneity is in good agreement with SEM and EDS analyses, which together suggest reduced particle clustering and a more uniform matrix in the pectin-containing formulation.

### 3.7. Stability Behaviour

The long-term storage stability of the optimized milk-based cocoa beverage was systematically monitored over a 9-month period by evaluating critical physicochemical parameters, including the creaming index, WHC, pH, sedimentation, apparent viscosity, and torque (Table 6).

During the initial phase of storage (months 1 to 3), the WHC remained statistically invariant, fluctuating marginally between 85.50% and 83.55% (*p* > 0.05). This initial stability indicates highly effective water immobilization facilitated by the intact pectin–protein network. However, a significant reduction in WHC became evident by month 5 (79.80%), followed by a continuous decline to 76.75% by the end of the 9-month period. This progressive deterioration suggests a time-dependent weakening and structural rearrangement of the macromolecular pectin–protein matrix. The gradual reduction in WHC observed during storage can be explained by the time-dependent rearrangement of pectin & casein complexes. Pectin confers stability to dairy-based dispersions via electrostatic adsorption onto casein micelles, thereby generating a kinetically trapped, stabilized network [40]. Over extended storage durations, these kinetically stabilized complexes are susceptible to structural reorganization, leading to a partial weakening of the network and reduced water immobilization. The marked decrease in WHC observed post-month 5 is a direct macroscopic reflection of these underlying progressive microstructural rearrangements.

Sedimentation values corroborated this biphasic stability profile. For the first two months, sedimentation remained statistically undetectable, confirming excellent initial dispersion stability. The onset of particle settling was recorded at month 3 (0.23%), which subsequently escalated to a statistically significant 2.25% by month 4. Post-month 6, sedimentation increased markedly, culminating at 14.50% by month 9. This substantial increase denotes a gradual loss of structural integrity and increased particle mobility, indicating that the steric stabilization provided by pectin is temporally finite. Similarly, the creaming index exhibited an early yet initially controlled elevation. A statistically significant rise (*p* < 0.05) was observed as early as the second month (1.15%) compared to the baseline value at month 1 (0.33%). This upward trend persisted steadily throughout the storage duration, featuring a pronounced acceleration after month 7 and reaching a final value of 5.13% at month 9. Throughout the entire 9-month evaluation, the pH (6.87–6.58) of the system remained remarkably stable, exhibiting no significant variations (*p* > 0.05). This robust pH stability confirms that the observed macroscopic destabilization phenomena were not associated with acidification or chemical instability but were primarily driven by physical restructuring processes.

Rheological monitoring unveiled a continuous, time-dependent escalation in apparent viscosity. A statistically significant increase materialized from month 3 onwards (75.88 mPa·s), with a drastic surge recorded post-month 7, ultimately peaking at 104.55 mPa·s by month 9. This profound increase may be attributed to structural or microstructural rearrangements in the pectin–casein system, enhanced particle & polymer interactions, or partial aggregation within the matrix. Specifically, the increase in viscosity during storage of pectin–casein complexes can be attributed to microstructural rearrangements that lead to closer packing of these complexes [28]. As the storage period progresses, the slow reorganization of the electrostatically bound pectin layers on the casein micelle surfaces can reduce the intermolecular distance between neighboring complexes, thereby amplifying interparticle interactions [41]. This closer, dense microstructural packing effectively restricts macroscopic particle mobility and increases resistance to flow, yielding a substantially higher apparent viscosity. Consequently, the reduction in the system’s free volume associated with this closer packing directly limits the matrix’s ability to immobilize water, fundamentally explaining the concurrent decrease in WHC and the acceleration of sedimentation during prolonged storage.

Torque measurements flawlessly mirrored the viscometric trends, exhibiting a progressive increase throughout the storage lifecycle. While torque values remained statistically constant during the first trimester (0.303–0.340 mN·m), a significant elevation commenced at month 5 (0.377 mN·m), culminating at 0.469 mN·m by month 9. These rheological and physical stability findings underscore the critical role of pectin as a potent kinetic stabilizer, capable of significantly delaying, rather than completely preventing, the inevitable long-term physical destabilization of complex dairy-based suspensions.

## 4. Conclusions

The development of cocoa-based dairy beverages was successfully conducted in two steps. In the first step, the beverage was produced at varying pectin concentrations (0.038–0.462%) and pressures (5.85–34.1 MPa). By considering parameters such as WHC, sedimentation, creaming index, Viscosity, and sensorial properties, the (0.4% pectin, 10 MPa) sample was selected as the optimal formula in the later step. This investigation highlights the synergistic potential of pectin concentration and homogenization pressure in enhancing the physical stability of beverages. According to results, pectin at 0.4% increased viscosity and WHC while reducing sedimentation and creaming by forming electrostatic networks with milk proteins, as evidenced by zeta potential, particle size, and molecular docking analyses. Homogenization at moderate pressures (10 MPa) promoted uniform cocoa dispersion and microstructural homogeneity, confirmed by SEM-EDS. The optimized formulation showed better sensory attributes (e.g., appearance, flavor, and mouthfeel) and 9-month storage performance, with gradual changes in viscosity and separation attributable to long-term pectin–protein rearrangements rather than complete destabilization. These results affirm pectin’s role as a natural alternative that can be successfully applied under industrial processing conditions to produce stable, homogeneous, and consumer-acceptable beverages. Future research could explore synergies between pectin and other hydrocolloids, or advanced processing techniques, to further mitigate long-term instabilities.

## Figures and Tables

**Table 1:** The response values obtained with the run points of the created CCRD experimental design.

Run	Pectin Concentration (%)	Homogenization Pressure (MPa)	WHC (%)	Sedimentation (%)	Creaming Index (%)	pH	Viscosity (mPa·s)	Torque (mN·m)
1	0.100	30.0	69.00 ± 1.41	18.29 ± 1.12	21.35 ± 1.36	6.85 ± 0.01	27.82 ± 0.53	0.125 ± 0.010
2	0.462	20.0	86.50 ± 0.71	n.d.	4.73 ± 0.16	6.84 ± 0.03	80.68 ± 1.11	0.362 ± 0.005
3	0.250	20.0	78.60 ± 0.85	8.96 ± 1.47	2.19 ± 0.16	6.85 ± 0.01	60.93 ± 1.34	0.274 ± 0.012
4	0.250	34.1	73.60 ± 3.39	14.29 ± 1.59	28.27 ± 0.82	6.83 ± 0.01	55.85 ± 0.78	0.198 ± 0.020
5	0.400	30.0	81.22 ± 1.10	5.50 ± 0.47	22.65 ± 0.71	6.89 ± 0.02	68.20 ± 1.00	0.296 ± 0.021
6	0.038	20.0	67.00 ± 0.99	20.04 ± 1.12	16.08 ± 0.65	6.87 ± 0.03	26.53 ± 0.50	0.119 ± 0.011
7	0.100	10.0	73.00 ± 1.41	13.58 ± 0.82	11.73 ± 0.71	6.88 ± 0.01	28.21 ± 0.61	0.126 ± 0.013
8	0.250	5.85	81.04 ± 0.65	3.54 ± 0.29	1.43 ± 0.06	6.85 ± 0.02	56.88 ± 0.26	0.241 ± 0.017
9	0.250	20.0	78.10 ± 2.69	7.08 ± 0.71	3.54 ± 0.65	6.86 ± 0.03	57.71 ± 0.30	0.254 ± 0.016
10	0.250	20.0	77.40 ± 0.85	6.79 ± 0.77	3.69 ± 0.22	6.88 ± 0.02	51.62 ± 0.40	0.232 ± 0.015
11	0.400	10.0	85.28 ± 0.40	1.25 ± 0.35	2.31 ± 0.33	6.89 ± 0.01	66.24 ± 0.80	0.287 ± 0.023
Model			Linear	Quadratic	Quadratic	No significant model	Linear	No significant model
*p* value			0.3782	0.3843	0.0830		0.0423	
R^2^			0.9871	0.9586	0.9747		0.9745	
Adjusted-R^2^			0.9839	0.9482	0.9493		0.9697	
Predicted- R^2^			0.9764	0.9261	0.8268		0.9469	

n.d.: not detected.

**Table 2:** Optimum point obtained from CCRD analysis.

Products	WHC (%)	Sedimentation (%)	Creaming Index (%)	pH	Viscosity (mPa·s)	Torque (mN·m)
Optimum predicted	86.17	n.d.	n.d.	6.88	69	0.294
Optimum experimental	86.04 ± 0.51	n.d.	n.d.	6.87 ± 0.02	67.39 ± 0.55	0.293 ± 0.014

n.d.: Not detected. Experimental values are expressed as mean ± standard deviation (SD) (*n* = 3). The optimum predicted values were generated by the response surface model.

**Table 3:** Predicted binding energies and non-covalent interactions between D-galacturonic acid (D-GalA) and the major milk proteins obtained from molecular docking.

Protein (Structure Source) ^1^	Binding Energy (kcal/mol)	Interaction Type	Residue	Protein Group Involved	H···A (Å)	D···A (Å)	Donor Angle (°)
α-Casein (I-TASSER homology model)	−4.2	H-bond	Gln170	Side-chain amide N–H (donor)	2.76	3.12	102.76
		Salt bridge	Arg166	Side-chain guanidinium with ligand carboxylate	–	4.13 ^2^	–
		Salt bridge	Arg166	Side-chain guanidinium with ligand carboxylate	–	4.49 ^2^	–
β-Casein (I-TASSER homology model)	−3.7	H-bond	Val216	Backbone C=O (acceptor)	2.00	2.93	174.81
		H-bond	Gly218	Backbone N–H (donor)	2.63	3.30	125.64
		H-bond	Phe220	Backbone C=O (acceptor)	2.24	2.90	123.95
		H-bond	Phe220	Backbone N–H (donor)	3.05	3.94	150.29
α-Lactalbumin (PDB ID 6IP9)	−5.3	H-bond	Glu49	Side-chain carboxylate O (acceptor)	2.16	3.11	168.28
		H-bond	Leu105	Backbone N–H (donor)	2.22	3.12	152.24
		H-bond	Ala106	Backbone N–H (donor)	2.25	3.23	169.48
β-Lactoglobulin (PDB ID 3NPO)	−6.3	H-bond	Trp19	Backbone C=O (acceptor)	2.28	2.79	111.78
		H-bond	Tyr42	Backbone C=O (acceptor)	2.76	3.34	119.29
		H-bond	Gln59	Side-chain amide N–H (donor)	2.20	3.14	160.20
		H-bond	Gln159	Side-chain amide C=O (acceptor)	3.48	3.79	101.27
		H-bond	Cys160	Backbone N–H (donor)	3.23	3.72	112.73
		H-bond	His161	Backbone N–H (donor)	3.17	3.98	141.19
		H-bond	His161	Side-chain imidazole N (acceptor)	2.71	3.14	108.09

^1^ Structures of α-casein (αs1-casein) and β-casein were generated by homology modelling (I-TASSER), whereas α-lactalbumin and β-lactoglobulin were retrieved from the RCSB Protein Data Bank. Binding energies were predicted with AutoDock Vina (PyRx 0.8) and interactions were assigned with the Protein–Ligand Interaction Profiler (PLIP). H-bond, hydrogen bond. H···A, hydrogen–acceptor distance. D···A, donor–acceptor distance. Donor angle, D–H···A angle. Donor and acceptor refer to the role of the protein group in the hydrogen bond. ^2^ Distance between the centroids of the charged groups (Arg166 guanidinium and D-GalA carboxylate).

**Table 4:** Analysis results of the optimum point sample.

Products	L*	a*	b*	Z-Ave (nm)	Zeta Potential (mV)
Optimum sample	48.22 ± 0.15	8.71 ± 0.02	8.84 ± 0.12	401.40 ± 8.79	−30.80 ± 0.49

The standard deviation is represented by ± values (*n *= 3).

**Table 5:** Elemental composition of milk-based cocoa beverages without hydrocolloid and the optimum formulation, determined by energy-dispersive X-ray spectroscopy (EDS).

Products	C (%)	N (%)	O (%)	Na (%)	*p* (%)	Cl (%)	K (%)	Ca (%)
No hydrocolloid	69.30 ± 0.15 ^a^	6.63 ± 0.83 ^a^	16.63 ± 0.18 ^b^	0.56 ± 0.06 ^a^	0.00 ^b^	1.38 ± 0.11 ^a^	3.79 ± 0.10 ^a^	1.72 ± 0.38 ^a^
Optimum sample	67.97 ± 1.70 ^a^	6.86 ± 2.30 ^a^	18.23 ± 5.03 ^a^	0.47 ± 0.12 ^b^	0.33 ± 0.01 ^a^	1.23 ± 0.05 ^b^	3.54 ± 0.43 ^a^	1.47 ± 0.08 ^b^

The standard deviation is represented by ± values (*n *= 3). Different letters in each column (according to Tukey test) are statistically significantly different (*p* < 0.05).

**Table 6:** Stability of optimum point sample.

Months	WHC (%)	Sedimentation Rate (%)	Creaming Index (%)	pH	Viscosity (mPa·s)	Torque (mN·m)
1	85.50 ± 0.71 ^a^	n.d.	0.33 ± 0.04 ^a^	6.87 ± 0.03 ^a^	67.55 ± 0.72 ^a^	0.303 ± 0.012 ^a^
2	84.60 ± 0.57 ^a^	n.d.	1.15 ± 0.21 ^b^	6.85 ± 0.01 ^a^	71.10 ± 2.26 ^a^	0.318 ± 0.009 ^a^
3	83.55 ± 0.64 ^a^	0.23 ± 0.03 ^a^	1.70 ± 0.28 ^b^	6.83 ± 0.02 ^a^	75.88 ± 2.79 ^b^	0.340 ± 0.011 ^a^
4	81.50 ± 0.71 ^ab^	2.25 ± 0.35 ^b^	2.05 ± 0.07 ^c^	6.79 ± 0.01 ^a^	80.68 ± 2.51 ^c^	0.361 ± 0.005 ^ab^
5	79.80 ± 0.42 ^b^	4.20 ± 0.28 ^c^	2.60 ± 0.14 ^c^	6.77 ± 0.01 ^a^	83.61 ± 2.08 ^c^	0.377 ± 0.008 ^b^
6	78.35 ± 0.64 ^b^	6.45 ± 0.64 ^d^	2.88 ± 0.11 ^cd^	6.71 ± 0.04 ^a^	88.67 ± 2.50 ^cd^	0.400 ± 0.007 ^b^
7	78.00 ± 1.41 ^b^	12.60 ± 0.85 ^e^	3.35 ± 0.21 ^d^	6.69 ± 0.02 ^a^	90.35 ± 1.91 ^d^	0.405 ± 0.009 ^b^
8	77.75 ± 0.35 ^bc^	13.30 ± 0.42 ^e^	4.20 ± 0.28 ^e^	6.63 ± 0.03 ^a^	97.40 ± 1.98 ^e^	0.438 ± 0.012 ^bc^
9	76.75 ± 1.77 ^c^	14.50 ± 0.71 ^e^	5.13 ± 0.18 ^f^	6.58 ± 0.02 ^a^	104.55 ± 0.98 ^e^	0.469 ± 0.013 ^c^

The standard deviation is represented by ± values (*n *= 3). Different letters in each column (according to Tukey test) are statistically significantly different (*p* < 0.05). n.d.: not detected. The samples were stored at 4 °C during storage.

## Data Availability

The original contributions presented in the study are included in the article, further inquiries can be directed to the corresponding authors.
